# An improved vitrification protocol for the fast and safe storage of mouse oocytes^†^

**DOI:** 10.1093/biolre/ioaf215

**Published:** 2025-09-22

**Authors:** Tina Hodgson, Hollie Lane, Helen Horsler, Juliette Horwood, Laura Denti, Fabio Delaqua, Katharine Crawley, Benjamin Davies

**Affiliations:** The Genetic Modification Service, The Francis Crick Institute, London, UK; The Genetic Modification Service, The Francis Crick Institute, London, UK; The Genetic Modification Service, The Francis Crick Institute, London, UK; The Genetic Modification Service, The Francis Crick Institute, London, UK; The Genetic Modification Service, The Francis Crick Institute, London, UK; The Genetic Modification Service, The Francis Crick Institute, London, UK; The Genetic Modification Service, The Francis Crick Institute, London, UK; The Genetic Modification Service, The Francis Crick Institute, London, UK

**Keywords:** oocytes, vitrification, cryopreservation, rederivation, in vitro fertilization

## Abstract

Cryopreservation methods for archiving and distributing mouse strains mostly focus on freezing embryos or sperm. Although protocols for the cryopreservation of wild-type mouse oocytes are available, these methods have not been widely adopted in large biomedical research facilities. This is partly due to a lack of validation of the available methods on a large scale using a range of genetically modified oocytes. Furthermore, some of the existing methods report a relatively low rate of fertilization, requiring either the zona pellucida to be physically breached or the inclusion of cumulus cells to enable efficient fertilization, interventions which might be incompatible with maintaining hygiene barriers. Existing methods also often use cryovials rather than straws or slimline vitrification devices, which are more practical for storage and handling. Here, we present a robust vitrification protocol for large-scale oocyte cryopreservation, achieving high viability and fertilization rates comparable to fresh oocytes. We have extensively tested the protocol for in vitro fertilization of many genetically altered strains, using both genetically altered and wild-type C57BL/6J oocytes and sperm. Providing an archive of cryopreserved oocytes harboring genetically modified alleles separately from archives of cryopreserved sperm allows multiple allele combinations to be generated during the rederivation process. This reduces subsequent breeding steps and the need to maintain live stocks of mice. Furthermore, cryopreserving oocytes for later use enables them to be obtained from females at the optimal age, thereby reducing the number of mice required and providing greater scheduling flexibility for subsequent genetic modification and rederivation work.

## Introduction

Genetically altered mice are widely used in biomedical science to explore gene function and serve as animal models of human genetic disease, facilitating the development of novel therapeutic and diagnostic tools. When working with mouse models, the 3R principles must be adhered to in order to reduce the number of mice that need to be bred and maintained. Cryopreservation of embryos and germplasm, along with assisted reproduction technologies, provides a convenient and practical means of achieving this by enabling the robust archiving of underused strains. Furthermore, these technologies facilitate collaboration as mouse lines can be exchanged easily between different facilities by transferring cryopreserved material. This avoids the negative welfare impacts and logistical difficulties of transporting live animals.

Cryopreservation of mouse embryos by slow-rate freezing is a commonly used technique, which allows gentle equilibration with cryoprotectants, preventing ice crystal formation [1]. Vitrification is a faster and more convenient cryopreservation technique, involving embryos being held in a high concentration of cryoprotectant solution, which can then be rapidly supercooled with liquid nitrogen (LN_2_), avoiding crystallization [2, 3]. However, the high concentrations of cryoprotectants and lack of osmotic equilibrium inherent with this method are embryo-toxic and strict storage below the glass transition temperature is essential [4].

Step-wise vitrification methods, where exposure to progressively higher concentrations of a cryoprotectant ameliorates some of the toxicity effects, have proved compatible with oocyte vitrification and subsequent in vitro fertilization (IVF) [5–10], even when using delicate C57BL/6J oocytes. However, these protocols have been tested only with wild-type and not with genetically modified oocytes, which may have varying attributes and levels of fragility, and so further testing is needed to assess their suitability for large-scale use in rederivation pipelines. Importantly, these protocols have been established for vitrification of oocytes in cryovials, rather than in straws or other slimline vitrification devices, the latter being more convenient for the archiving of large-scale strain repositories. A step-wise vitrification protocol is available at the European Mutant Mouse Archive (EMMA) [11], which uses straw storage; however, this protocol is for vitrification of 2-cell-stage embryos and subsequently further optimization of a vitrification method suitable for oocytes that utilizes straw storage at scale is needed. Finally, one of these protocols reported only reliable IVF rates when oocytes were vitrified as cumulus–oocyte complexes [5, 6], which could present biosafety issues when storing the samples in liquid-phase nitrogen.

A newer technique of “equilibrium” vitrification has further mitigated many of these issues [12, 13] and has been validated for straw storage. Ficoll PM70 is added to the vitrification solution—this macromolecule helps displace water, allowing the vitrification media to solidify with lower concentrations of cryoprotectants. In addition, sucrose is added to help embryo dehydration and to reduce the amount of cryoprotectant present in the embryos. This combination allows the embryo to be in osmotic balance or “equilibrium,” which helps prevent crystallization and therefore devitrification during transient warming. Initially established for embryo vitrification using relatively high concentrations of cryoprotectants (35% ethylene glycol), a subsequent modification reduced the concentration of cryoprotectants to 20% using a 1:1 mix of ethylene glycol and dimethyl sulfoxide [14, 15]. These milder conditions allowed the vitrification of oocytes and were compatible with subsequent warming and IVF [16]. Despite this improvement, fertilization was only achievable when the zona pellucida was mechanically disrupted, which would preclude the use of such embryos for rederivation into an SPF barrier facility, and was only validated at small scale (four to eight oocytes per straw).

We present here an optimized equilibrium vitrification protocol which is suitable for large-scale archiving of oocytes in devices that can utilize straw storage for use in substantial cryopreservation and rederivation pipelines. The method is compatible with oocytes generated via hyperovulation from genetically modified strains and is compatible with efficient IVF using processes that do not compromise hygiene barriers. High rates of fertilization are shown for a range of genetically modified strains with side-by-side comparisons with freshly prepared oocytes presented.

## Materials and methods

**Figure 1:**
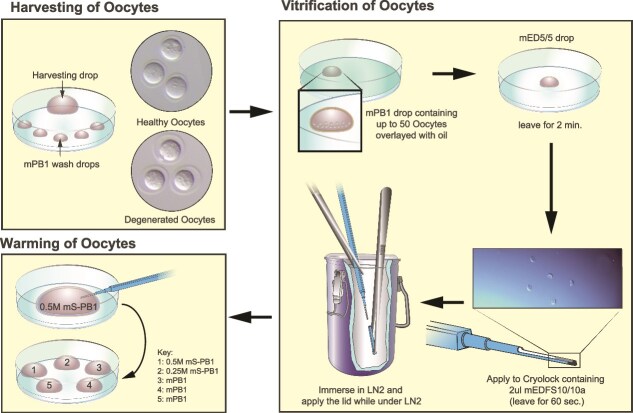
Visual overview of the oocyte vitrification method.

An overview of the vitrification method is shown in Figure 1, all the materials are listed in Table 1, and all the reagents and media are outlined in Table 2.

**Table 1:** List of equipment needed

Equipment/Materials	Company	Catalogue number
0.22 μm filter	Sartorius	10509821
0.45 μm filter	Sartorius	11786953
10 μl pipette tips 200 μl pipette tips 1000 μl pipette tips	StarLabs StarLabs StarLabs	S1121-2710 S1122-1730 S1122-1730
35 mm culture dish	Falcon	351008
60 mm culture dish	Corning	30622013
7 ml bijou tube	DWK	327145
Cryo-gloves	Merck life science UK Ltd	Z183512
Cryolock devices	Biotech	CL-R-CT
LN_2_ dewar	KGW Isotherm	9032726
Forceps	Philip Harris	B8R00176
Glass capillaries	World Precision Instruments	TW100-6
Heatpad	OKOLAB	H401-T-Controller
Incubator/warm box	Cooper Surgical	K59500
Mouth aspiration device	Merck Life Science UK Ltd	A5177-5EA
P10 pipette	Gilson	F144055M
P1000 pipette	Gilson	F144059M
P200 pipette	Gilson	F144058M
Watchmaker forceps	Fine Science Tools	91150-20

**Table 2:** List of media and media composition (masses of components are listed for an example media volume (shown in italics))

Media/Solutions	Final conc.	Supplier	Catalogue number	Example
mPB1				*100 ml*
NaCl	136 mM	Merck Life Science UK Ltd	S5886	800 mg
KCL	2.68 mM	Merck Life Science UK Ltd	P5405	20 mg
KH_2_PO_4_	1.47 mM	Merck Life Science UK Ltd	P5655	20 mg
MgCl_2_.6H_2_O	491.9 μM	Merck Life Science UK Ltd	M2393	10 mg
Na_2_HPO_4_	8.1 mM	Merck Life Science UK Ltd	S5136	115 mg
Na-pyruvate	327.7 μM	Merck Life Science UK Ltd	P4562	3.6 mg
Glucose	5.55 mM	Merck Life Science UK Ltd	G6152	100 mg
Penicillin	201.4 μM	Merck Life Science UK Ltd	P7794	7.5 mg
Streptomycin	68.62 μM	Merck Life Science UK Ltd	S1277	5.0 mg
Bovine serum albumin	45.11 μM	Thermo Fisher Scientific	AM2616	300 mg
Fetal bovine serum	20%	Thermo Fisher Scientific	A5256701	20 ml
Sterile filter 0.22 μm Keep at 4°C for up to a month				
mS-PB1				*10 ml*
mPB1 Sucrose	0.5 M	Merck Life Science UK Ltd	S1888	10 ml 1.71 g
Filter 0.45 μm Keep at 4°C for same expiry as mPB1				
mFSa				*10 ml*
mPB1 Sucrose Ficoll PM70	0.5 M 30% (w/v)	Merck Life Science UK Fisher Scientific UK Ltd	S1888 11,500,734	7 ml 1.71 g 3 g
Vortex and leave overnight to dissolve Following day make up to 10 ml with mPB1				
mED5/5				*6 ml*
mPB1 Ethylene glycol Dimethyl sulfoxide	5% 5%	VWR international Ltd Merck Life Science UK Ltd	03750 D2650	5400 μl 300 μl 300 μl
Filter day after making using 0.22 μm Keep at 4°C for up to 6 months Protect from light with foil				
mEDFS10/10a				*6 ml*
mFSa Dimethyl sulfoxide Ethylene glycol	80% 10% 10%	Merck Life Science UK Ltd VWR international Ltd	D2650 03750	4800 μl 600 μl 600 μl
Filter 0.45 μm Keep at 4°C for up to 6 months Protect from light with foil				
Hyaluronidase solution	10 mg/ml			*3 ml*
Hyaluronidase mPB1		Merck life science UK Ltd	H4272	30 mg 3 ml
EmbryoMax HTF		Merck Life Science UK Ltd	Mr-70-D	
Sage oil		Cooper Surgical	27,854	
Chorulon 1500 IU,	50 IU/mL	MWI animal health	09020051	
CARD HyperOva 5x1 ml		2BScientific	KYD-010-EX-X5	

### Animals

Animal procedures were performed in accordance with the UK Home Office Animal (Scientific Procedures) Act 1986 under project license PP6551133. Animals were housed in individually ventilated cages and provided with food and water ad libitum, maintained on a 12 h/12 h light cycle (150–200 lux). Typically, wild-type C57BL/6J female mice or genetically modified female mice, predominantly on a C57BL/6J genetic background, at 21–35 days of age, were hyperovulated as previously described [17]. 2-cell embryos were surgically transferred into the oviducts of pseudopregnant CD1 recipients at 0.5 days post coitum and, in side-by-side comparisons between vitrified and fresh oocytes, an equal number of embryos were transferred.

*NOTE: For the efficient generation of oocytes using a small number of donor females, hormonal stimulation through the administration of gonadotropins or inhibin antisera is recommended.*

### Harvesting of oocytes

*NOTE: All movement of oocytes within these methods is performed with a capillary, controlled with either a mouth aspiration device or suitable alternative.*

1. Dissect oviducts.
2. Per pair of oviducts, pipette a 100 μl mPB1 harvesting drop and 5× 100 μl mPB1 wash drops onto a 60 mm dish (Figure 1).
3. Add two oviducts to each mPB1 drop and release the cumulus mass from the oviduct by tearing the ampulla with forceps, gently drag the cumulus mass out of the oviduct into the mPB1, and discard the harvested oviduct tissue.
4. Add 2 μl hyaluronidase solution to each 100 μl mPB1 drop containing the cumulus mass.
5. When the cumulus cells start to dissipate (approximately 1–2 min), collect the oocytes and wash through 5× 100 μl mPB1 drops.
6. Select only the healthy oocytes for vitrification (see Figure 1 for examples)

*NOTE: The process can be paused here for up to 90 min. Oocytes can go into a pre-equilibrated HTF/20% fetal bovine serum (FBS) dish in the incubator (37*°C*, CO_2_, 5% O_2_) for up to 90 min before the vitrification steps.*

### Vitrification of oocytes

1. Preparation steps:
  1. Remove all the solutions from the fridge 30 min before use, to ensure they are at room temperature.
  2. Label the Cryolock devices with a suitable code that identifies the oocyte strain/donor mouse.
  3. Prepare a small dewar or box of LN_2_.
  4. Prepare a wash dish by flooding a 35 mm dish with mPB1.
2. Each Cryolock holds up to 50 oocytes. Count the oocytes and prepare a dish with 1× 50 μl mPB1 drop per Cryolock, overlaid with mineral oil. Transfer the groups of up to 50 oocytes into their mPB1 drops where they can wait until ready to vitrify.
3. Prepare one 50 μl drop of mED5/5 on a 35 mm dish for each of the drops of mPB1 you have.
4. Vitrifying one Cryolock at a time: add 2 μl of mEDFS10/10 to cover approximately one-third of the Cryolock device tip and set aside.
5. Transfer a group of up to 50 oocytes from their mPB1 drop into an mED5/5 drop. Start a count-up timer.
6. Collect the oocytes (in an absolute minimum volume of media, i.e., <0.1 μl) in a capillary, ready to expel into the 2 μl drop of mEDFS10/10a on the Cryolock when the timer reaches 2 min.
7. Expel the oocytes into the media on the Cryolock at 2 min, use your capillary to mix the oocytes with the media, and then plunge the Cryolock directly into the LN_2_ when the timer reaches 3 min (so oocytes are in mEDFS10/10a for 1 min).
8. Using forceps and cryo-safe gloves, cool the lid of the Cryolock in the LN_2_. When the LN_2_ has displaced all of the air in the lid, the lid can be put onto the Cryolock, making sure the tip with the oocytes and the lid remain under LN_2_ at all times.
9. Wash your glass capillary in the mPB1 wash dish to remove vitrification media before moving on to the next batch of oocytes.
10. Repeat steps 4–9 until all the oocytes are vitrified.

### Warming of oocytes

*NOTE: The EMMA IVF protocol* [11]*, based on the CARD IVF method* [18]*, is used following the warming of the oocytes.*

1. Preparation steps:
  1. Remove all the solutions from the fridge 30 min before use, to ensure they are at room temperature before use.
  2. Prepare IVF fertilization dishes according to the EMMA IVF protocol [11].
  3. Prepare a wash dish by flooding a 35 mm dish with HTF (as this is the media used for the EMMA IVF protocol [11]) and put it into the incubator (37°C, 5% CO_2_, 5% O_2_).
  4. Prewarm a bijou tube of mS-PB1 (1.2 ml per Cryolock to warm) to 37°C.
  5. Quickly remove Cryolock devices from storage and put immediately into a small dewar of LN_2_.
  6. Warm up the heat pad.
2. Per Cryolock being warmed, prepare a 60 mm dish with a 100 μl drop of 0.5 M mS-PB1, a 100 μl drop of 0.25 M mS-PB1 and 3× 100 μl drops of mPB1. Place it on the 37°C heat pad.
3. Add 1 ml prewarmed 0.5 M mS-PB1 to a separate 60 mm dish and place it on the heat pad.
4. Remove the lid of the Cryolock under LN_2_ and then plunge the tip of the Cryolock straight into the 1 ml prewarmed mS-PB1 on the dish; gently waft the Cryolock in the solution for 10 s to ensure all the embryos are removed.
5. Start a count-up timer.
6. Start collecting oocytes and transfer them into the 100 μl 0.5 M mS-PB1 drop prepared for them and leave them in this drop on the heat pad until the timer reaches 5 min.
7. Transfer the oocytes into the 0.25 M mS-PB1 drop and leave them there until the timer reaches 10 min.
8. Wash through the three drops of mPB1, leaving them in the final drop until the timer reaches 15 min.
9. Wash through the flooded HTF dish into the pre-prepared fertilization dish for IVF and leave to incubate for a minimum of 30 min before adding sperm.
10. Continue with the IVF procedure as with fresh oocytes, according to the EMMA IVF protocol [11].

### Statistics

Statistical comparisons of the fertilization rates, birth rates, viability rate on warming, and blastocyst development rate were performed using a logistic regression model. Each result was assigned indicator variables to label whether vitrified or fresh oocytes were used and which genetically modified line was used. The oocytes used (vitrified or fresh) were treated as fixed effects and the genetic lines as random effects. The model was then fitted using the function glmer (family = binomial) from the R package lme4. Data sets were presented using Graphpad Prism software v10.1.2.

## Representative results

A previous equilibrium vitrification method [16] was only tested for small-scale freezing and was found not to be suitable for large-scale rederivation projects, as the zona pellucida needed to be manually breached. To overcome this requirement and develop a more robust vitrification protocol suitable for large-scale archiving and rederivation, several changes were implemented to this protocol, summarized below:

- A calcium-free media was adopted to help reduce any increases in intracellular calcium during the cryopreservation process.
- FBS at 20% was included to help prevent zona hardening and increase oocyte survival.
- Vitrification was performed in Cryolock devices, which facilitated the use of minimal volumes and hence increased the warming and cooling rate when compared to straw cryopreservation substantially.

The protocol was extensively tested for the vitrification of C57BL/6J oocytes followed by warming and IVF using both fresh and frozen genetically modified sperm. In parallel, IVF was also performed using fresh oocytes with the same sperm sample to allow a direct comparison. Following IVF, the resulting 2-cell embryos were surgically transferred into pseudopregnant recipient mice to assess the in vivo development and birth-rate.

**Figure 2:**
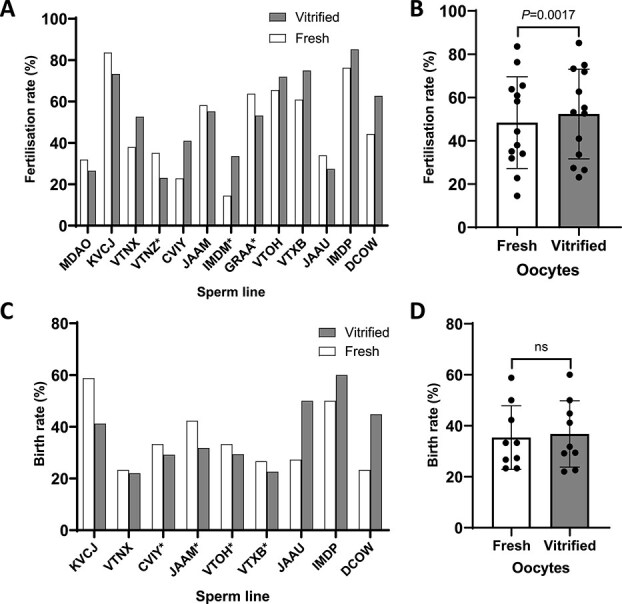
Fertilization and transfer rates of vitrified/warmed C57BL/6J oocytes compared to freshly harvested oocytes. (A) Graph showing fertilization rates (number of 2-cell embryos following overnight culture as a fraction of total oocytes used for IVF) using sperm from different genetically modified lines. (B) Histogram showing the distribution of the data in A. (C ) Graph showing embryo transfer birth rate (the number of live born as a percentage of the number of embryos transferred) for the different genetically modified lines. Data from embryo transfers that involved 2-cell embryos undergoing additional slow-rate freezing and subsequent thawing are marked with “*.” (D) Histogram showing the distribution of the data in (C). In all cases, the individual genetically modified lines are distinguished by an in-house four-letter code. Vitrified/warmed data is shown in shaded columns; data from fresh control embryos is shown in unshaded columns.

Overall, survival of vitrified C57BL/6J oocytes postwarming was high (99.3% survival, Supplementary Table S1), indicating that the vitrification method did not compromise viability. Fertilization rates following IVF using vitrified and warmed oocytes were comparable with those obtained with fresh oocytes (Figure 2A and B), and, when analyzed across all genetic alleles, revealed a significant increase (*β* = −0.27, *P* = 0.0017) in fertilization. The subsequent birth rates following embryo transfer of the fertilized embryos were also comparable, with no significant difference between the two groups (*β* = −0.068, *P* = 0.72; Figure 2C and D).

**Figure 3:**
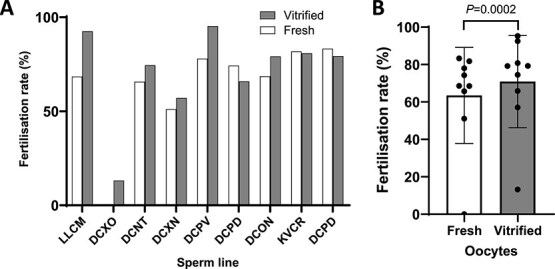
Fertilization rates of vitrified/ warmed genetically modified oocytes compared to freshly harvested oocytes. (A) Graph showing fertilization rates of genetically modified oocytes (number of 2-cell embryos following overnight culture as a fraction of total oocytes used for IVF) using sperm from different genetically modified lines. (B) Histogram showing the distribution of the data in (A). In all cases, the individual genetically modified lines are distinguished by an in-house four-letter code. Vitrified/warmed data is shown in shaded columns; data from fresh control embryos is shown in unshaded columns.

In addition, the protocol was tested for the vitrification of genetically modified C57BL/6J oocytes followed by warming and IVF with fresh genetically modified sperm. Survival postwarming was 100% (Supplementary Table S1) and, again, fertilization rates following IVF using vitrified and warmed oocytes exceeded those obtained with fresh oocytes (*β* = −0.51, *P* = 0.0002; Figure 3A and B), confirming that the vitrification protocol is sufficiently robust to be used for the cryopreservation of genetically modified oocytes.

**Figure 4:**
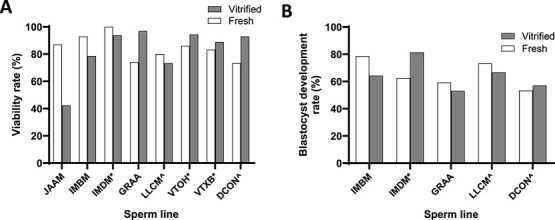
Viability and in vitro development of 2-cell embryos thawed after slow-rate freezing. Two-cell embryos derived from fertilized vitrified/warmed and freshly harvested oocytes were frozen again by slow-rate freezing. The frozen 2-cell embryos were then thawed and the percentage of viable embryos (A) and the development to the blastocyst stage (B) are shown. Viable embryos were those appearing unchanged after the freeze/thawing process, with normal blastomeres without fragmentation, lysis, or other signs of degeneration. In all cases, the individual genetically modified lines are distinguished by an in-house four-letter code. Vitrified/warmed data is shown in shaded columns; data from fresh control embryos is shown in unshaded columns. Samples fertilized with frozen sperm are shown with “*”; samples using genetically modified oocytes are marked with “^.”

The protocol was also tested by generating 2-cell embryos from vitrified and warmed oocytes via IVF and cryopreserving the resulting embryos using a conventional slow-rate freezing method [19]. The survival upon thawing showed some variation in outcome, but overall, no significant difference was found when using 2-cell embryos generated by IVF of fresh oocytes versus warmed vitrified oocytes (*β* = 0.25, *P* = 0.351; Figure 4A), In addition, the subsequent development of these embryos in vitro to the blastocyst stage showed no difference between the two groups (*β* = −0.062, *P* = 0.844; Figure 4B) and they developed to term when transferred to pseudopregnant recipient mice (Figure 2C).

## Troubleshooting

### Transfer of media between solutions

Critical to the success of this method are the small volumes of media on the Cryolock. Subsequently, it is very important to only transfer the absolute minimum amount of media possible alongside the oocytes when transferring them from the mED5/5 to the mEDFS10/10a on the Cryolock (<0.1 μl). Transferring large amounts of mED5/5 into the mEDFS10/10a will significantly affect the end concentrations of the cryoprotectants and other ingredients in the media, which could influence the survival and development of the oocytes when warmed. If the viability of the warmed oocytes is lower than expected (usually close to 100%; Supplementary Table S1), then this could be an area to investigate to ensure only minimal volumes are being transferred.

### Bubbles in media

When adding media to the Cryolock and when transferring oocytes to the mEDFS10/10a on the Cryolock, it is important not to introduce any bubbles to the media. The presence of bubbles in the viscous media would lead to embryos sticking to the bubbles and not properly equilibrating before being submerged in LN_2_.

### Handling of the Cryolock devices

It is important to handle the Cryolock devices with care, in particular when putting the lid on under LN_2_. It must be ensured that the LN_2_ has stopped evaporating around the Cryolock and the lid before the lid is put on, and that the liquid drop containing the oocytes remains visible. If the recovery rate of the oocytes is lower than expected (usually close to 100%; Supplementary Table S1), then oocytes may be being lost from the end of the Cryolock when putting the lid on under LN_2_.

It is equally important to be careful when taking the lid off when warming. The lid should be fully clear of the end of the Cryolock before taking the Cryolock out of LN_2_; if the end of the Cryolock gets caught on the lid, it is possible that the end of the Cryolock could snap.

### Appearance of the oocytes

The oocytes change in appearance throughout this protocol. When added to mED5/5, the resulting dehydration is visible, and when added to the mEDFS10/10a the oocytes then take on a glassy sickle-shaped appearance (Figure 1). When the oocytes are being warmed, they can appear as if they are lysing in the first sucrose solution. All these appearances are normal, and upon completion of the protocol, the oocytes should appear healthy (Figure 1).

## Discussion

Large biomedical research facilities maintaining genetically modified mouse models require cryopreservation techniques for secure archiving of lines for both business continuity and distribution needs. In addition, it is well established that cryopreservation techniques represent an important 3R, reduction, since lines that are infrequently used can be eliminated as live colonies [20].

The majority of facilities achieve this through the cryopreservation of sperm samples, fertilized zygotes, or other preimplantation-stage embryos. Surprisingly, cryopreservation of oocytes is infrequently performed in large facilities. Despite the availability of several protocols, anecdotally the methods have proved challenging to establish and many of the protocols are insufficiently validated across a spectrum of genetically modified strains.

We present here a robust protocol for vitrification of oocytes in Cryolock devices, allowing straw storage, which is compatible with high rates of oocyte recovery and efficient IVF, without a requirement for any physical breach of the zona pellucida or the presence of cumulus cells. We present data that validates the protocol extensively across multiple genetically modified C57BL/6J strains, importantly with side-by-side data from fresh oocytes—high rates of IVF and production are maintained.

Compared with existing protocols, the modifications that contributed toward the optimization of this technique are as follows:

- Calcium-free media: During the vitrification process, there is an increase of intracellular calcium due to the use of cryoprotectants such as DMSO and ethylene glycol, which, as well as increasing the likelihood of triggering cell apoptosis [21], leads to exocytosis of cortical granules and subsequent hardening of the zona [22], preventing sperm from fertilizing the oocyte. The use of calcium-free media helps suppress this rise in intracellular calcium and prevents zona hardening and cell death [5].
- Addition of FBS: Following reports that FBS increased oocyte survival [6, 23], fertilization, and blastocyst development rates, 20% FBS was added to all our media. The presence of FBS helps extrude cryoprotectants from the perivitelline space of embryos and also prevents zona hardening due to its fetuin content [24].
- Use of Cryolock devices: Cryolock devices allow the amount of vitrification media used to be minimized to 2 μl, increasing the cooling and warming rate of the oocytes substantially and thus minimizing oocyte damage [25]. According to the manufacturer’s validation, a cooling rate of 19,800°C/min and a warming rate of 27,500°C/min are achieved when using Cryolock devices, which are at a level known to protect against cellular damage when using low concentrations of cryoprotectants [26]. Importantly, Cryolock devices can be stored as straws, allowing large-scale archiving with minimal storage space, when compared with storage in cryovials. As an alternative to Cryolock devices, alternative in-house-made vitrification devices have recently been reported [27].

The most commonly used vitrification protocol is a stepwise method known as the “DAP213 method” on account of the 2 M DMSO, 1 M acetamide, and 3 M propylene glycol cryoprotectant solution used [9], which is widely used for the cryopreservation of 2-cell embryos [11]. Our protocol, based on equilibration vitrification techniques [12, 13], uses lower concentrations of cryoprotectant solution (10% [1.28 M] DMSO and 10% [1.61 M] ethylene glycol) and thus may be more suitable for delicate developmental stages, such as the oocyte. Finally, the use of lower concentrations of cryoprotectants, alongside the water displacement achieved by including Ficoll PM70 and sucrose in the vitrification solution, means that our protocol, in contrast with DAP213-based methods, does not require the oocytes to be precooled or handled on a block cooler or vitrification cooling plate. In addition, the maintenance of samples at extremely low temperatures becomes less critical for equilibrium vitrification methods, where vitrified embryos have been shown to be viable when stored at −80°C for several days [16].

Somewhat surprisingly, the overall IVF rates were actually higher when using oocytes that had been vitrified, compared with fresh oocytes. We believe this may be a consequence of the greater scrutiny used to assess the health of the oocytes prior to vitrification, since only the best-quality oocytes were used for vitrification. In addition, it could also be that FBS within the vitrification media softens the zona pellucida, making fertilization more efficient. An increase in parthenogenetic activation of vitrified oocytes may also have contributed to this increase, although the equal birth rates observed following surgical transfer of a matched number of embryos between the vitrified and fresh groups suggest this is unlikely to be occurring at an appreciable level.

Establishment of oocyte vitrification within facilities has several important advantages, and all of these have significant 3R impact, contributing to a reduction in the number of live animals that need to be maintained.

Firstly, the technique provides greater flexibility of combining genetically modified alleles. Both gamete types can be independently stored and subsequently combined when live mice are needed or embryos of specific genotypes are required for further genetic manipulation.

Secondly, it can be a useful tool when managing mouse colonies for cryopreservation. When breeding mice to obtain sufficient numbers for embryo cryopreservation, females of the desired genotype may be generated at insufficient numbers. Vitrification of the oocytes when the female mice are at an optimal age allows more efficient IVF and minimizes maintenance of live mice. The IVF can then be performed at a later date, when sufficient oocytes are available, and thus the superovulation of females of suboptimal age is avoided.

Thirdly, banks of oocytes from wild-type and other commonly used strains such as Cre and Flp recombinase strains can be generated and these used directly for rederivations using imported genetically modified sperm. This eliminates the need to keep live colonies of the recipient strain, reducing the number of live mice that need to be maintained.

In conclusion, adapting previous vitrification methods to suit large-scale vitrification of oocytes has provided a valuable tool in rederivation and cryopreservation pipelines and has the potential to reduce mouse usage substantially. The lack of differences in fertilization and transfer rates between fresh and vitrified oocytes lends confidence to the robustness of this protocol.

## Supplementary Material

Supplementary_Table_1_ioaf215

## Data Availability

The data underlying this article are available in the article and in its online supplementary material.
