# Supplementary material for: An improved vitrification protocol for the fast and safe storage of mouse oocytes
Source: Biol Reprod. 2025 Sep 22;113(6):1355–63. doi: 10.1093/biolre/ioaf215 (PMC12706468; doi:10.1093/biolre/ioaf215)
Supplement: Supplementary_Table_1_ioaf215 [file supplementary_table_1_ioaf215.docx]

| **Oocyte group** | **Contents** | **Recovered** | **Recovery rate** | **Viable** | **Viability rate** |
| --- | --- | --- | --- | --- | --- |
| **C57BL/6J oocytes** | 1142 | 1129 | 98.9% | 1121 | 99.3% |
| **GM oocytes** | 579 | 579 | 100.0% | 579 | 100.0% |

Supplementary Table 1: Recovery and viability rates of vitrified/thawed C57BL/6J and genetically modified oocytes. Recovery rate (number of oocytes recovered from the Cryolocks divided by the number of oocytes vitrified) and viability rate (number of oocytes which survive the warming process divided by the number of oocytes vitrified), of both C57BL/6 and genetically modified oocytes. Oocytes with an appearance that is unchanged from that prior to vitrification, with cytoplasm without blebbing or fragmentation, are scored as having survived the warming process.
